# Functional assessment of the V390F mutation in the CCTδ subunit of chaperonin containing tailless complex polypeptide 1

**DOI:** 10.1007/s12192-021-01237-x

**Published:** 2021-10-15

**Authors:** Josefine Vallin, Julie Grantham

**Affiliations:** Department of Chemistry and Molecular Biology, University of Gothenburg, 40530 Gothenburg, Sweden

**Keywords:** Molecular chaperone, Cancer cell line, Chemotaxis, GrowDex®

## Abstract

The chaperonin containing tailless complex polypeptide 1 (CCT) is a multi-subunit molecular chaperone. It is found in the cytoplasm of all eukaryotic cells, where the oligomeric form plays an essential role in the folding of predominantly the cytoskeletal proteins actin and tubulin. Both the CCT oligomer and monomeric subunits also display functions that extend beyond folding, which are often associated with microtubules and actin filaments. Here, we assess the functional significance of the CCTδ V390F mutation, reported in several cancer cell lines. Upon transfection into B16F1 mouse melanoma cells, GFP-CCTδ^V390F^ incorporates into the CCT oligomer more readily than GFP-CCTδ. Furthermore, unlike GFP-CCTδ, GFP-CCTδ^V390F^ does not interact with the dynactin complex component, p150^Glued^. As CCTδ has previously been implicated in altered migration in wound healing assays, we assessed the behaviour of GFP-CCTδ^V390F^ and other mutants of CCTδ, previously used to assess functional interactions with p150^Glued^, in chemotaxis assays. We developed the assay system to incorporate a layer of the inert hydrogel GrowDex® to provide a 3D matrix for chemotaxis assessment and found subtle differences in the migration of B16F1 cells, depending on the presence of the hydrogel.

**Supplementary Information:**

The online version contains supplementary material available at 10.1007/s12192-021-01237-x.

## Introduction

Chaperonin containing tailless complex polypeptide 1 (CCT) is a molecular chaperone that promotes the folding of substrates in an ATP-dependent manner. In its oligomeric state, CCT is composed of two rings stacked back-to-back where each ring contains eight individual subunits (named α-θ or 1–8), each occupying a fixed position within the ring (reviewed by Vallin and Grantham 2019). The eight CCT subunit genes are essential in yeast (reviewed by Stoldt et al. 1996), and CCT is found in the cytoplasm of all eukaryotes. All CCT subunits share a common domain architecture, consisting of a highly conserved equatorial domain containing an ATP-binding pocket, a less conserved apical substrate binding domain and a linker domain.

The CCT oligomer folds the cytoskeletal proteins actin and tubulin, which are both dependent on CCT to reach their final native structure (Sternlicht et al. 1993) and its folding activity also extends to other proteins, although not all are considered to be obligate folding substrates (Willison 2018). In addition to folding, CCT oligomer has functions that can be connected to assembled cytoskeletal structures, for example, by interacting with and potentially sequestering the actin filament severing protein gelsolin (Brackley and Grantham 2011; Svanstrom and Grantham 2016). Furthermore, the CCT oligomer binds to the transcription factor STAT3, potentially modulating STAT3 phosphorylation (Vallin et al. 2021).

The CCT oligomer is dynamic with regard to assembly (Roobol et al. 1999a), and in addition to CCT existing as an oligomer, it can also be detected as smaller assemblies (Collier et al. 2021; Liou and Willison 1997) and as a pool of free monomers. Equivalent mutants in the ATP-binding site of all eight CCT subunits give rise to different phenotypes in yeast consistent with the possibility of CCT subunits having distinct monomer functions (Amit et al. 2010). Furthermore, in yeast, a non-stoichiometric level of CCT subunits is observed, also consistent with at least some CCT subunits being available in increased levels as monomers (Matalon et al. 2014). For some CCT subunits, individual monomeric functions have indeed been established, for example, in cell lysates, CCTα, γ, ζ, and θ have been shown to co-assemble with microtubules (Roobol et al. 1999b). Furthermore, monomeric CCTε can interact with the co-transcriptional activator of the serum response factor pathway, MRTF-A (Elliott et al. 2015), and CCTδ can localize to the plasma membrane via interactions with p150^Glued^, a component of the dynactin complex (Echbarthi et al. 2018).

CCT levels are known to be upregulated in hepatocellular and colonic carcinoma samples (Yokota et al. 2001) and in other human tumour samples (The Cancer Genome Atlas TCGA). CCT2 (CCTβ) has also been implicated in cell growth and invasion (Showalter et al. 2020) and depletion of CCTβ, CCTδ, or CCTζ by siRNA lead to a reduction in growth (Grantham et al. 2006). In addition, the gene expression of CCTδ and CCTγ subunits has been shown to be increased in cells from a primary tumour migrating into a needle of extracellular matrix (Wang et al. 2004). As gene expression will be deregulated in tumours, changes to the CCT subunit levels will have the potential to lead to changes in the availably of CCT subunits both for assembly/folding and for individual CCT subunit monomer functions. Here, we study the point mutation V390F in CCTδ, which occurs in five human cancer cell lines: carcinomas of the oesophagus, ovary, stomach, aerodigestive tract, and haematopoietic/lymphoid neoplasm (reported in COSMIC) and is located at the border between the intermediate and apical domain. We characterize this mutation using the mouse melanoma B16F1 cell line and assess the potential impact of CCTδ^V390F^ upon CCT oligomer assembly, interactions with p150^Glued^, and in chemotaxis migration.

## Results and discussion

### Enhanced incorporation of GFP-CCTδ^V390F^ into the CCT oligomer

**Fig. 1:**
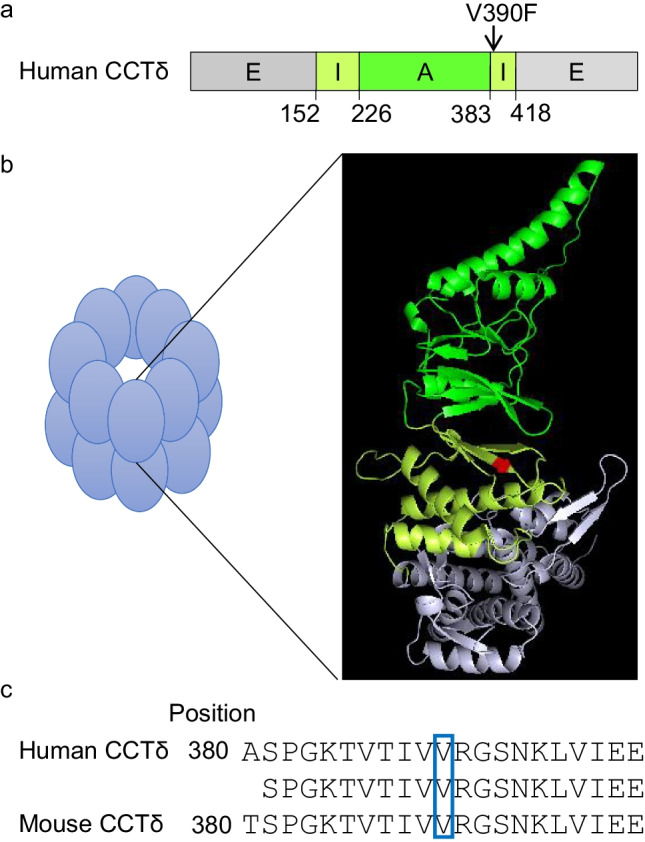
Position of the highly conserved CCTδ^V390^. **a** Cartoon representation of the CCT domains (grey E equatorial, yellow I intermediate, green A apical) with domain boundaries and V390 indicated. **b** Cartoon of the CCT oligomer and the structure of the thermosome alpha chain (PDB: 1A6D) are shown where the domains are colour-coded as in **a** and the position of V390 indicated in red. **c** Sequence alignment of human and mouse CCTδ in the region close to V390

The CCTδ^V390F^ mutation is located at the border between the apical and intermediate domains (Fig. 1a) and is shown mapped onto the structure of the alpha thermosome chain (PDB: 1A6D) (Fig. 1b). This region of sequence is identical between human and mouse CCTδ (Fig. 1c). The corresponding position is well conserved between orthologues, and comparison of subunits within the same species shows this position in other subunits to be occupied by leucine, isoleucine, or valine; all hydrophobic and non-polar amino acids (Sup. Figure 1a and b).

We assessed if the V390F substitution could have an impact upon CCT oligomer assembly in B16F1 mouse melanoma cells. To this end, we transfected cells with either CCTδ or CCTδ^V390F^ (using the mouse sequence) as GFP fusions or with a myc-tag. Fusing a GFP-tag to the N-terminus of CCT subunit constructs has been shown previously to hinder incorporation into the CCT oligomer (Spiess et al. 2015). However, a C-terminal myc-tag appears to be more tolerated with regard to assembly (Brackley and Grantham 2010).

**Fig. 2:**
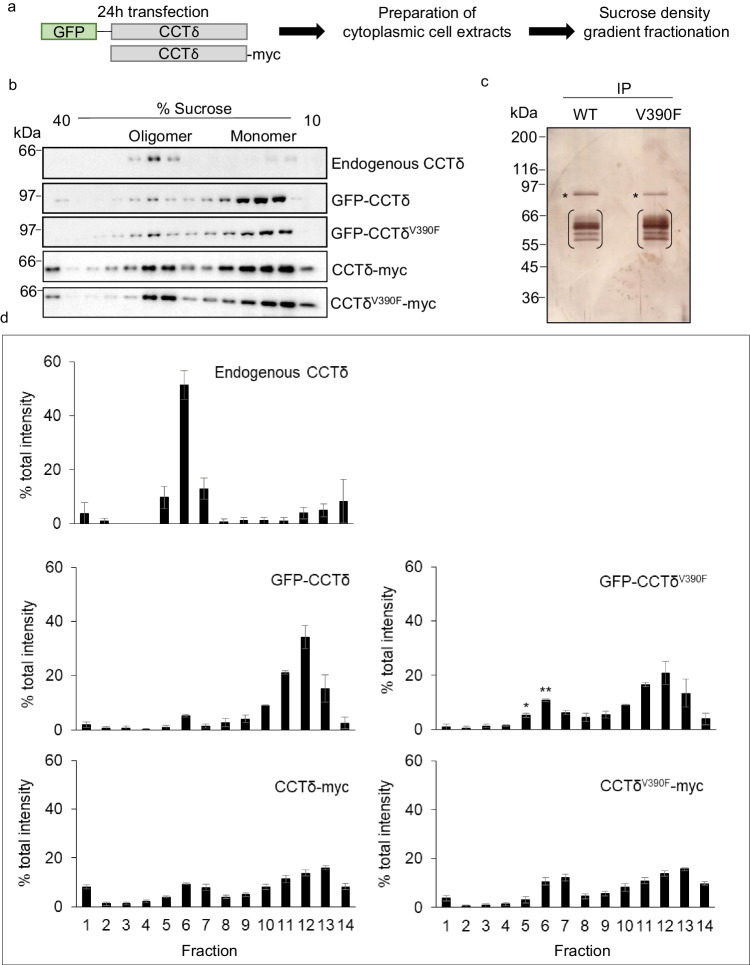
The V390F mutation in CCTδ may promote CCT oligomerization. **a** Schematic representation of experimental procedures for analysis of constructs in B16F1 cells and illustration of the GFP and myc-tag positions. **b** Representative sucrose gradient profiles of endogenous CCTδ, GFP-CCTδ, GFP-CCTδ^V390F^, CCTδ-myc, and CCTδ^V390F^-myc. **c** Immunoprecipitation of the CCT oligomer-containing fractions using anti-GFP nanobodies analyzed by SDS PAGE and silver staining. The GFP-CCT fusions are marked with an asterisk and the position of endogenous CCT subunits indicated with brackets. **d** Intensity analysis for three experiments as shown in **b**. Percent of intensity for each fraction was compared to the same set of transfections (GFP-CCTδ^V390F^ to GFP-CCTδ, and CCTδ^V390F^-myc to CCTδ-myc). Student *t*-test is used where significant when *p < 0.05, ** *p* < 0.01, *n* = 3

Lysates from transfected B16F1 cells were analyzed by sucrose density gradient fractionation to separate the CCT oligomer from monomeric subunits after transfection with constructs with either a GFP fusion or a myc-tag (Fig. 2a). Profiles of endogenous CCTδ, GFP-CCTδ, and GFP-CCTδ^V390F^ were then analyzed by Western blotting (Fig. 2b). For GFP-CCTδ and GFP-CCTδ^V390F^, the fractions corresponding to the position of the CCT oligomer were further analyzed by immunoprecipitating the GFP fusions with anti-GFP nanobodies. In both cases, endogenous CCT subunits were found to co-precipitate, consistent with low levels of the GFP fusions assembling with endogenous CCT subunits (Fig. 2c). The sucrose gradient profiles were then analyzed by densitometry. The endogenous CCTδ profile is consistent with most of CCTδ being incorporated in the oligomer (Fig. 2d, top panel). In addition to the B16F1 cells shown here, profiles of endogenous subunits have also been examined in the BE colon carcinoma cell line (Grantham et al. 2006) and balb3T3 mouse fibroblast cell line (Brackley and Grantham 2010) where the oligomeric form is abundant. As expected, the addition of a GFP-tag to the N-terminus of CCTδ hindered GFP-CCTδ oligomerization and most of GFP-CCTδ migrated in the lighter fractions of the sucrose gradient (Fig. 2d, middle left panel). However, in comparison to GFP-CCTδ, GFP-CCTδ^V390F^ was significantly enriched in the CCT oligomer-containing fractions (Fig. 2d, middle right panel). Thus, a single amino acid substitution, from valine to phenylalanine, and thereby the addition of an aromatic side chain in CCTδ can override the robust method of hindering oligomer incorporation by the addition of a GFP-tag to the N-terminus. We then assessed the effect of the V390F substitution when expressed as CCTδ^V390F^-myc and observed a slight, although not statistically significant, enhancement of CCTδ^V390F^-myc in the oligomeric fractions (Fig. 2d, lower panels). The Western blot signals for endogenous CCTδ and the GFP and myc constructs from fractions corresponding to the oligomer and monomer peaks in the sucrose gradient constructs were combined and assessed as a percentage of the total signal (Table 1). This analysis reflects that of the individual sucrose gradient profiles shown in Fig. 2d. As CCTδ-myc is readily able to oligomerize, differences between the mutant and wild-type constructs may be less apparent, as enhanced oligomerization by V390F may then be limited by the levels of the endogenous subunits.

**Table 1:** Western blot signals corresponding to the oligomer and monomer fractions were calculated as a percentage of the total signal across the gradient profile. In all cases, the oligomer was considered to be in fractions 5–7 and the free monomer pool was considered to be fractions 12–14 for endogenous and myc-tagged, and 11–13 for GFP fusions. Standard error of the mean ( ±) is calculated from the percentage of the total signal for oligomer or monomer fractions (11–13 or 12–14 and 5–7, respectively) from three individual experiments (*n* = 3)

	Oligomer	Monomer
Endogenous CCTδ	73.97% ± 6.14	17.05% ± 10.3
GFP-CCTδ	7.49% ± 1.76	70.75% ± 1.72
GFP-CCTδ^V390F^	22.17% ± 1.17	50.77% ± 2.37
CCTδ-myc	21.10% ± 1.93	37.94% ± 2.89
CCTδ^V390F^-myc	25.57% ± 1.24	39.05% ± 0.73

### The V390F substitution abrogates the binding of CCTδ to p150^Glued^

**Fig. 3:**
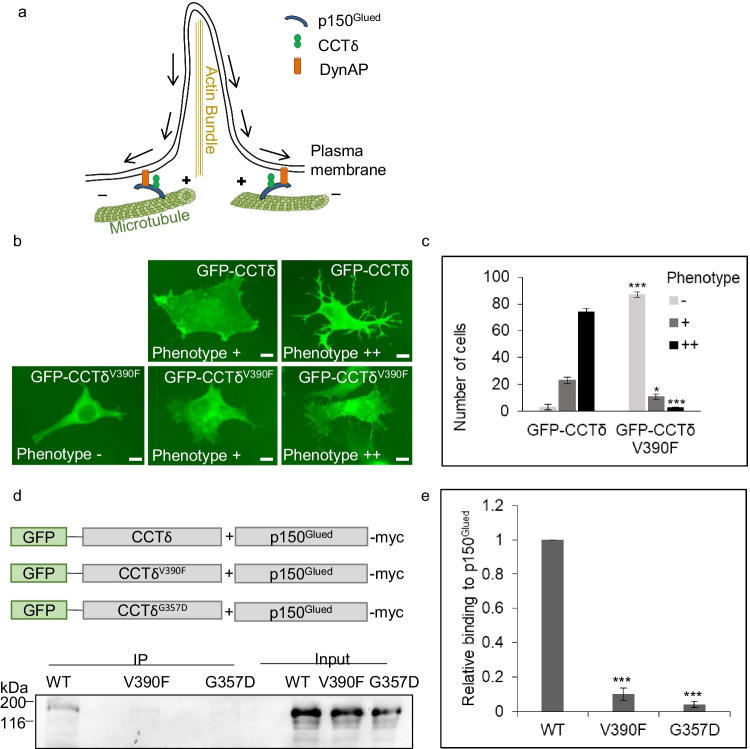
GFP-CCTδ^V390F^ does not bind to p150^Glued^. **a** Illustration of an inward movement of the plasma membrane induced by monomeric CCTδ binding to p150^Glued^ as described in Echbarthi et al. (2018). **b** Wide-field microscopy images of B16F1 cells transfected with GFP-CCTδ or GFP-CCTδ^V390F^. Scale bars correspond to 10 μm. **c** Quantification of cells scored for having no ( −), weak ( +), or strong (+ +) protrusion phenotype. Three biological replicates were performed and 100 cells from each counted. The graph shows the mean from the three experiments and Student *t*-test was used comparing WT to V390F (* < 0.05 and *** < 0.001). **d** The combinations of constructs used for immunoprecipitation are illustrated. Immunoprecipitation with anti-GFP nanobodies from lysates of cells co-transfected with p150^Glued^-myc and GFP-CCTδ, GFP-CCTδ^V390F^, or GFP-CCTδ^G357D^ analyzed by Western blotting using anti-myc antibody. **e** Analysis of three biological replicates of **d** are shown. For each individual experiment, the signal from the IP is normalized to input and WT relative binding is set to 1. The relative binding for the two mutations is compared to WT. The graph shows the mean of the three experiments. Student *t*-test was used to compare the mutations to WT (*** < 0.001), *n* = 3

When levels of CCTδ monomer are increased by transfection, CCTδ translocates to the plasma membrane and there is an induction of actin containing cell surface protrusions, which we refer to as a ‘protrusion phenotype’ (Spiess et al. 2015). This phenotype was used to study functions of monomeric CCTδ and we found that the p150^Glued^ component of the dynactin complex binds to CCTδ (Echbarthi et al. 2018). Together with the transmembrane protein dynAP, p150^Glued^ is required for the formation of the cell surface protrusions by creating an inward movement of the plasma membrane along microtubules (Echbarthi et al. 2018 and illustrated in Fig. 3a).

Two points of mutations within CCTδ, D104E and G357D, prevent the formation of the protrusion phenotype and do not translocate to the plasma membrane (Spiess et al. 2015). D104E is an ATP-binding pocket mutation, and GFP-CCTδ^D104E^ has been shown to bind to p150^Glued^, but does not induce cell surface protrusions, whilst the apical domain mutation G357D abolishes binding to p150^Glued^ and therefore cells expressing GFP-CCTδ^G357D^ do not display the protrusion phenotype (Echbarthi et al. 2018). Consistent with p150^Glued^ binding to the dynein motor and mediating minus end–directed microtubule transport, we demonstrated that the protrusion phenotype does not form when cells are treated with the microtubule-depolymerizing drug, nocodazole (Echbarthi et al. 2018). We have therefore hypothesized that CCTδ binds via its apical domain to p150^Glued^ and, via its ATPase activity, may enhance the binding of p150^Glued^ to dynAP at the plasma membrane (Echbarthi et al. 2018).

To study if increased expression of CCTδ harbouring the V390F mutation can induce the protrusion phenotype, B16F1 cells were transfected with GFP-CCTδ or GFP-CCTδ^V390F^ and scored as having high, low, or no protrusion phenotype upon analysis by wide-field microscopy (Fig. 3b). Quantification from three biological replicates demonstrates there is a substantial reduction in the number of cells with a protrusion phenotype in GFP-CCTδ^V390F^-expressing cells compared to cells expressing GFP-CCTδ (Fig. 3c). We then assessed if GFP-CCTδ^V390F^ is able to interact with p150^Glued^. Immunoprecipitation using GFP nanobody beads following co-transfection of p150^Glued^-myc with GFP-CCTδ, GFP-CCTδ^V390F^, or GFP-CCTδ^G357D^ (as a negative control) indicated no binding of GFP-CCTδ^V390F^ to p150^Glued^-myc (Fig. 3d and e), consistent with the expression of GFP-CCTδ^V390F^ not inducing a protrusion phenotype. It is important to note that whilst some GFP-CCTδ^V390F^ will be incorporated into the CCT oligomer, a substantial amount remains monomeric (Fig. 2d and Table 1); thus, it is unlikely that the loss of interaction with p150^Glued^ is due to limiting levels of monomeric GFP-CCTδ^V390F^.

Since CCTδ^V390F^ has a wild-type apical domain, we speculate that either V390 contributes to binding to p150^Glued^, or that the V390F substitution affects the conformation of the CCTδ apical domain. The latter possibility is consistent with the position of V390 being at the boundary between the apical domain and the flexible intermediate domain, which is responsible for inducing conformational changes from the equatorial domain to the apical domain.

### Assessment of cells expressing CCTδ mutations using chemotaxis assays with and without GrowDex® 3D matrix

Levels of GFP-CCTδ have previously been shown to affect cell migration, where cells having a moderate expression level of GFP-CCTδ show increased migration in wound healing assays in comparison to cells expressing GFP-CCTβ, GFP-CCTδ^G357D^, or GFP-CCTδ^D104E^ (Echbarthi et al. 2018). As this increased, directed cell migration is observed only where increased levels of monomeric CCTδ can bind to p150^Glued^ and have a functional ATPase activity, this implicates the CCTδ:p150^Glued^ interaction at the plasma membrane in affecting cell migration (Echbarthi et al. 2018).

To extend our previous studies, here we used a Boyden chamber trans-well assay where we incorporate a layer of gel matrix and use laminin as a chemoattractant to assess the migration of cells in a more 3D environment using B16F1 cells transfected with GFP-CCTδ, GFP-CCTδ^V390F^, GFP-CCTδ^D104E^, or GFP-CCTδ^G357D^.

**Fig. 4:**
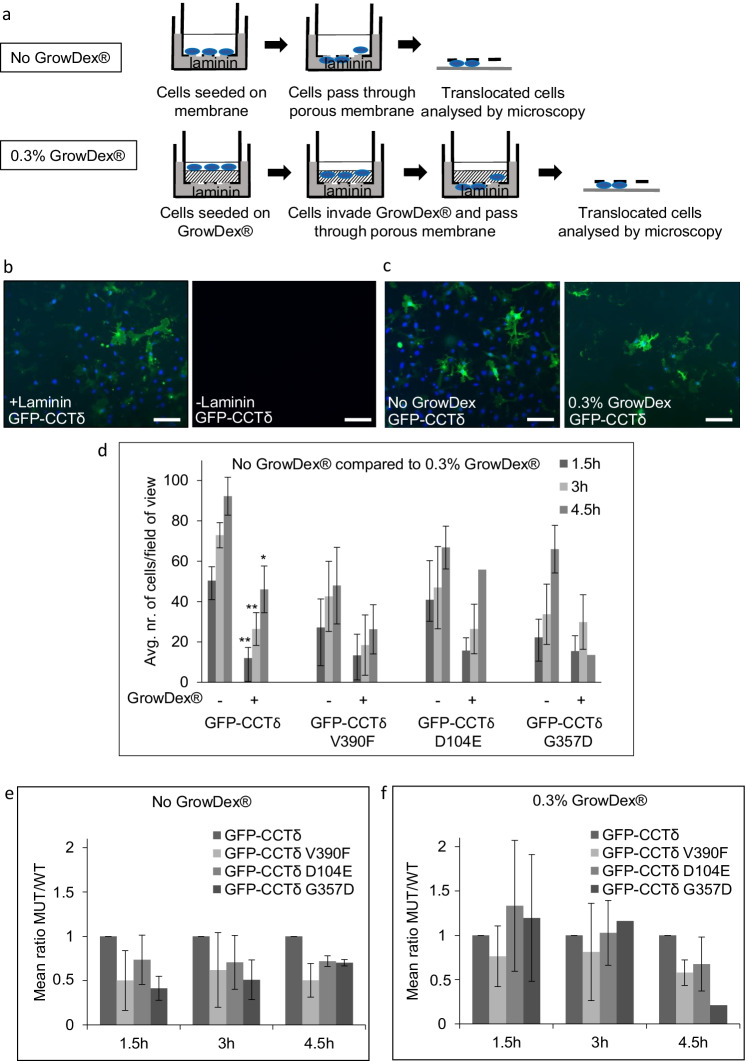
Effects of CCTδ mutations on chemotaxis migration. **a** Schematic overview of the experimental chemotaxis procedure where cells migrate through a porous membrane towards a chemoattractant with and without a layer of 0.3% GrowDex® matrix. **b** Wide-field microscopy images of cells migrating through a Boyden chamber trans-well membrane with and without laminin. **c** Representative wide-field microscopy images showing the experimental procedure described in **a**. **d** Comparison of the amount of cells migrating towards laminin through the Boyden chamber trans-well membrane compared to cells migrating towards laminin through the membrane after they have migrated through 0.3% GrowDex® 3D matrix layer. Student *t*-test is used where significant when *p < 0.05, **p < 0.01. **e** Chemotaxis migration in the absence of GrowDex® where the ratio between cells expressing GFP-CCTδ^V390F^, GFP-CCTδ^D104E^, and GFP-CCTδ^G357D^ and cells expressing GFP-CCTδ at three time points is calculated. GFP-CCTδ is set to 1. **f** Ratio between chemotaxis migration as described above for cells migrating through 0.3% GrowDex®. For each set of experiments in **d**, **e**, and **f**, *n* is between 2 and 5 where statistical analysis only has been performed where *n* ≥ 3. Scale bars correspond to 100 μm

Cells were either seeded directly onto the Boyden chamber membrane or onto a layer of GrowDex® cellulose-containing hydrogel (Fig. 4a). The use of GrowDex® is beneficial as it enables us to use laminin as a chemoattractant, compared to animal-based gels, which already contain laminin. Initially, we confirmed that cells only translocated through the membrane of the Boyden chamber in the presence of laminin (Fig. 4b). We then scored the numbers of transfected cells translocating through the membrane in the presence and absence of GrowDex®. Representative images of GFP-CCTδ expressing cells seeded directly onto the Boyden chamber or onto a layer of GrowDex® are shown in Fig. 4c. Cells included in the analysis were those that had completely translocated through the pores of the membrane and those in the process of translocating through the membrane.

Consistent with GrowDex® providing a substantial 3D matrix, there is a statistically significant reduction the number of GFP-CCTδ expressing cells able to migrate through the trans-well membrane in the presence GrowDex® 3D matrix compared to GFP-CCTδ expressing cells seeded in the absence of GrowDex® (Fig. 4d). For cells expressing GFP-CCTδ^V390F^, GFP-CCTδ^D104E^, or GFP-CCTδ^G357D^, there was no statistical difference in the numbers of cells translocating through the membrane in the presence and absence of GrowDex®, although the overall trend indicated a slight decrease in the number of translocating cells in the presence GrowDex® (Fig. 4d).

Next, to compare chemotaxis migration in the absence of GrowDex® between GFP-CCTδ and GFP-CCTδ^V390F^, GFP-CCTδ^D104E^, and GFP-CCTδ^G357D^, the numbers of cells translocating through the membrane for each of the mutations were compared to GFP-CCTδ transfected cells. The mean number of translocating cells was compared between GFP-CCTδ and GFP-CCTδ^V390F^, GFP-CCTδ^D104E^, and GFP-CCTδ^G357D^, and the ratio between GFP-CCTδ and GFP-CCTδ^V390F^, GFP-CCTδ^D104E^, and GFP-CCTδ^G357D^, where GFP-CCTδ is set to 1, was calculated (Fig. 4e). Although not statistically significant, there is a trend of the chemotaxis movement being reduced in cells expressing GFP-CCTδ^V390F^, GFP-CCTδ^D104E^, or GFP-CCTδ^G357D^ in comparison to cells expressing GFP-CCTδ (Fig. 4e). In contrast, when the same experiment was performed with a layer of 0.3% GrowDex®, a reduction in the number of migrating cells for GFP-CCTδ^V390F^, GFP-CCTδ^D104E^, or GFP-CCTδ^G357D^ expressing cells compared to GFP-CCTδ during chemotaxis up to 3 h was not observed (Fig. 4f).

To confirm that the differences observed were not a consequence of transfection efficiency, we calculated the transfection efficiencies and observed that this is in fact lower for GFP-CCTδ compared to that of GFP-CCTδ^V390F^, GFP-CCTδ^D104E^, and GFP-CCTδ^G357D^. For example, in one biological replicate, the transfection efficiencies were GFP-CCTδ = 65%, GFP-CCTδ^V390F^ = 75%, GFP-CCTδ^D104E^ = 72%, and GFP-CCTδ^G357D^ = 68%. The reduction in chemotaxis movement here is consistent with the observations of Echbarthi et al. (2018), where cells expressing GFP-CCTδ^D104E^ or GFP-CCTδ^G357D^ migrated more slowly in a wound healing assay compared to cells expressing GFP-CCTδ.

None of the mutations used here are able to induce the protrusion phenotype, which we consider to be a consequence of CCTδ monomer binding to p150^Glued^ and the ATPase activity of CCTδ inducing translocation to the plasma membrane (Echbarthi et al. 2018). This would suggest that the higher levels of cell migration observed for cells expressing GFP-CCTδ could be a consequence of increased interactions with p150^Glued^. The observations here that GFP-CCTδ is not faster through 0.3% GrowDex® compared to the three mutants suggest that the interaction between monomeric CCTδ and p150^Glued^ could potentially have a greater impact on certain types of cell motility. It is also possible that there is a difference in how the cells respond to laminin between the CCTδ constructs tested.

## Discussion

**Fig. 5:**
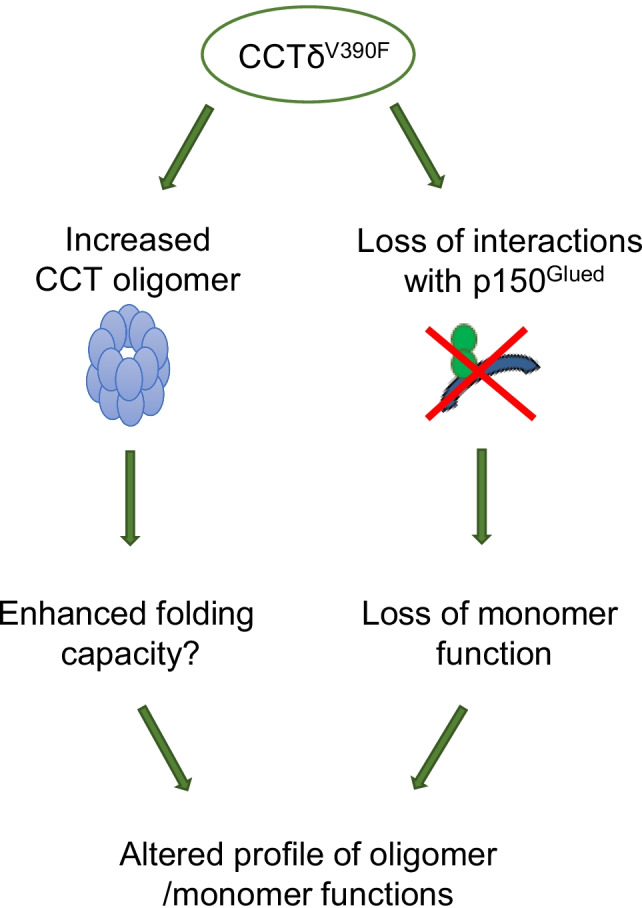
Consequences of CCTδ V390F mutation. Schematic figure to show that V390F mutation in CCTδ could result in two separate effects in the cell, increased CCT oligomer, and loss of CCTδ monomer function. Affecting CCT assembly or monomer-specific interactions would both result in an altered profile of oligomer and monomer functions

Here, we have assessed the CCTδ^V390F^ mutation with regard to oligomer assembly and monomeric function. We demonstrate that this mutation may promote assembly of the CCT oligomer, and as a monomer is unable to bind p150^Glued^. Thus, there are potentially two distinct consequences for cells harbouring the V390F mutation: increased levels of CCT oligomer and loss of p150^Glued^-mediated CCTδ monomer function (Fig. 5). The former may increase the folding capacity of the cell, whilst the latter will potentially reduce the possibility of certain types of cell migration. More assembled CCT oligomer would support an enhanced growth rate via the folding of actin and tubulin and other CCT folding substrates required for cell cycle progression, such as Cdc20 (Camasses et al. 2003). Indeed, CCT levels have previously been linked to cell growth (Yokota et al. 1999), and when CCT levels are depleted by siRNA, a decrease in cell growth is observed (Grantham et al. 2006).

CCT subunits can undergo gain of function/amplification in cancer and have, for example, been suggested to support breast cancer cell growth (Ghozlan et al. 2021; Xu et al. 2021). However, the effects of mutations of CCT subunits are less clear. It is possible that some mutations, such as the V390F substitution reported here, affect oligomerization and will thus have the potential effect in the cell in the same way as an increase in CCT expression levels and lead to an increased folding capacity/cell growth rates. The extent of CCT oligomer assembly will always be limited by the abundance of the other subunits, as they all exist in equal amounts in the assembled oligomer. Also, enhancement of oligomerization will potentially deplete the CCT monomer pool, reducing the availability of free subunits to perform monomeric-specific functions, such as CCTε binding to MRTF-A (Elliott et al. 2015). Furthermore, point mutations may interrupt specific binding interactions, as is the case for the V390F mutation with regard to p150^Glued^ binding. Thus, the effects of mutations and changes in CCT subunit expression may have complex consequences for the cell.

Mutations affecting assembly will have the potential to not only increase the folding capacity of the cell, but may also influence the availability of CCT oligomer for functions beyond folding such as modulating STAT3 activity (Vallin et al. 2021) and gelsolin activity (Svanstrom and Grantham 2016) and promoting the assembly of the von Hippel-Lindau tumour suppressor protein and Elongins (Melville et al. 2003).

Mutations in CCT subunits and changes in expression levels are not only relevant for cancer cell biology; for example, Brehme et al. (2014) report reductions in CCT expression during ageing and some neurodegenerative diseases. Furthermore, the C450Y mutation in CCTδ associated with hereditary sensory neuropathies may display reduced assembly (Sergeeva et al. 2014). Thus, the consequences of mutations in CCT subunits and changes in CCT expression levels, together with how CCT assembly is regulated, remain important questions for understanding the role of CCT in health and disease.

## Methods

### Cell culture

B16F1 cells were maintained in complete DMEM media (DMEM media (GIBCO Life Technologies) supplemented with 100 U/ml penicillin–streptomycin (GIBCO Life Technologies), 2.5 μg/ml Plasmocin (InvivoGen), and 10% heat-inactivated FBS (Invitrogen)) at 37 °C, 5% CO_2_.

### Transfection

GFP-CCTδ, GFP-CCTδ^D104E^ and GFP-CCTδ^G357D^ plasmid preparation is described by Spiess et al. (2015) and p150^Glued^ plasmid preparation is described by Echbarthi et al. (2018). The GFP-CCTδ^V390F^ plasmid was prepared using *QuickChange*™ site-directed mutagenesis cloning method using GFP-CCTδ as the template. B16F1 cells were transfected using Opti-MEM (GIBCO Life Technologies) together with Lipofectamine 2000 (Invitrogen).

### Sucrose density gradient fractionation

One day post-transfection, cells were detached from the cell culture dish by addition of 1 mM EDTA in PBS and washed twice in ice-cold PBS. Cells were then lysed in ice-cold lysis buffer (50 mM HEPES pH 7.2, 90 mM KCl, 0.5% IGEPAL, and 1/500 mammalian protease inhibitor (Sigma-Aldrich)) and post-nuclear supernatants obtained by centrifugation at 7000 rpm for 5 min at 4 °C in a benchtop centrifuge. Samples were loaded onto sucrose gradients containing a gradient of 40–10% sucrose in 50 mM HEPES pH 7.2, 90 mM KCl. Samples were then centrifuged at 4 °C for 18 h at 85,000* g* using a Beckman SW55 Ti rotor. Fourteen equal volume fractions were collected and resolved by SDS PAGE, before being transferred to a nitrocellulose membrane and analyzed by Western blotting.

### Immunofluorescence

On the day before transfection, B16F1 cells were seeded on glass coverslips that were pre-coated with laminin (Sigma-Aldrich). Cells were transfected on the glass coverslips, and the day after transfection, cells were washed once in complete PBS (supplemented with 1 mM CaCl_2_ and 0.5 mM MgCl_2_) and incubated for 10 min in 4% formaldehyde in complete PBS. Fixed cells were washed three times in PBS and with a final wash in MilliQ water before being mounted onto microscope slides using ProLong®Gold (Invitrogen). Wide-field images were taken using a Zeiss Axioplan microscope with AxioVision software.

### Immunoprecipitation

For immunoprecipitation, B16F1 cells were transfected, and the day after transfection, post-nuclear supernatants were prepared as described above. An additional centrifugation at 13,000 rpm for 2 min at 4 °C in a benchtop centrifuge was performed before incubation with GFP-trap®_A beads (Chromotek) for 1 h at 4 °C on a rotating wheel at 9 rpm. After incubation, beads were washed three times in lysis buffer and vacuum-dried before the addition of 1 × SDS sample buffer. Samples were resolved by SDS PAGE, before being transferred to a nitrocellulose membrane and analyzed by Western blotting.

### Boyden chamber migration

B16F1 cells were grown in normal cell culture conditions and transfected as described above. The next day, cells were detached as described above and diluted to give a final concentration of 130 × 10^4^ cells/ml. The Boyden chamber membrane inserts (TREVIGEN) were hydrated by addition of 100 μl DMEM media and were incubated in the cell culture incubator (37 °C, 5% CO_2_) for at least 30 min. GrowDex® (UPM Biomedicals) was dispensed from the syringe and weighed. DMEM media was added to give a final concentration of 0.3% w/v GrowDex® and mixed for 90 s to give an even distribution of GrowDex® in the tube. For membranes used for 3D migration studies, the 100 μl of DMEM incubated with the membrane was replaced by 100 μl 0.3% GrowDex®. Fifty microliters of cell suspension (130 × 10^4^ cells/ml) were added on top of the 100 μl DMEM or GrowDex® and moved to a well containing a 1/40 dilution of laminin in DMEM (Sigma-Aldrich L2020) to form the attractant gradient. Inserts were incubated in the cell culture incubator for 1.5, 3, or 4.5 h before fixing in 4% formaldehyde in complete PBS for 10 min. Inserts were washed 3 times in PBS, and the cells remaining on the upper side of the membrane were cleaned away using a cotton swab. Cells were then stained with 1 μg/ml DAPI (Thermo Scientific) and washed 3 times in PBS, and then, membranes were cut out from the insert and mounted onto a glass microscope slide using ProLong®Gold (Invitrogen). Wide-field images were taken using a Zeiss Axioplan microscope with AxioVision software.

### Sequence alignment

Sequences for mouse and human CCT subunits were obtained from UniProt and sequences were aligned using BLAST to compare the location of the V390 position in CCTδ to other subunits. The sequence of the thermosome was also aligned using BLAST, and the position corresponding to the CCTδ mutation mapped onto the structure of thermosome alpha chain (obtained from PDB: 1A6D) by using PyMol.

## Supplementary Information

Below is the link to the electronic supplementary material.

Supplementary Fig. 1 Sequence alignments of the human and mouse sequences of CCT subunits close to the position V390 in human CCTδ. (PPTX 100 KB)

Supplementary Fig. 2 Representative fields of view are shown of B16F1 cells transfected with either GFP-CCTδ or GFP-CCTδV390F. For both constructs one image taken at × 20 magnification (scale bar 100 μm) and two images taken at × 40 magnification (scale bar 10 μm) are shown. (PPTX 716 KB)

## Data Availability

Available on request.
