# Supplementary material for: Functional assessment of the V390F mutation in the CCTδ subunit of chaperonin containing tailless complex polypeptide 1
Source: Cell Stress Chaperones. 2021 Oct 15;26(6):955–64. doi: 10.1007/s12192-021-01237-x (PMC8578507; doi:10.1007/s12192-021-01237-x)
Supplement: Supplementary file 1 — Supplementary Fig. 1 Sequence alignments of the human and mouse sequences of CCT subunits close to the position V390 in human CCTδ. (PPTX 100 KB) [file 12192_2021_1237_MOESM1_ESM.pptx]

## Slide 1
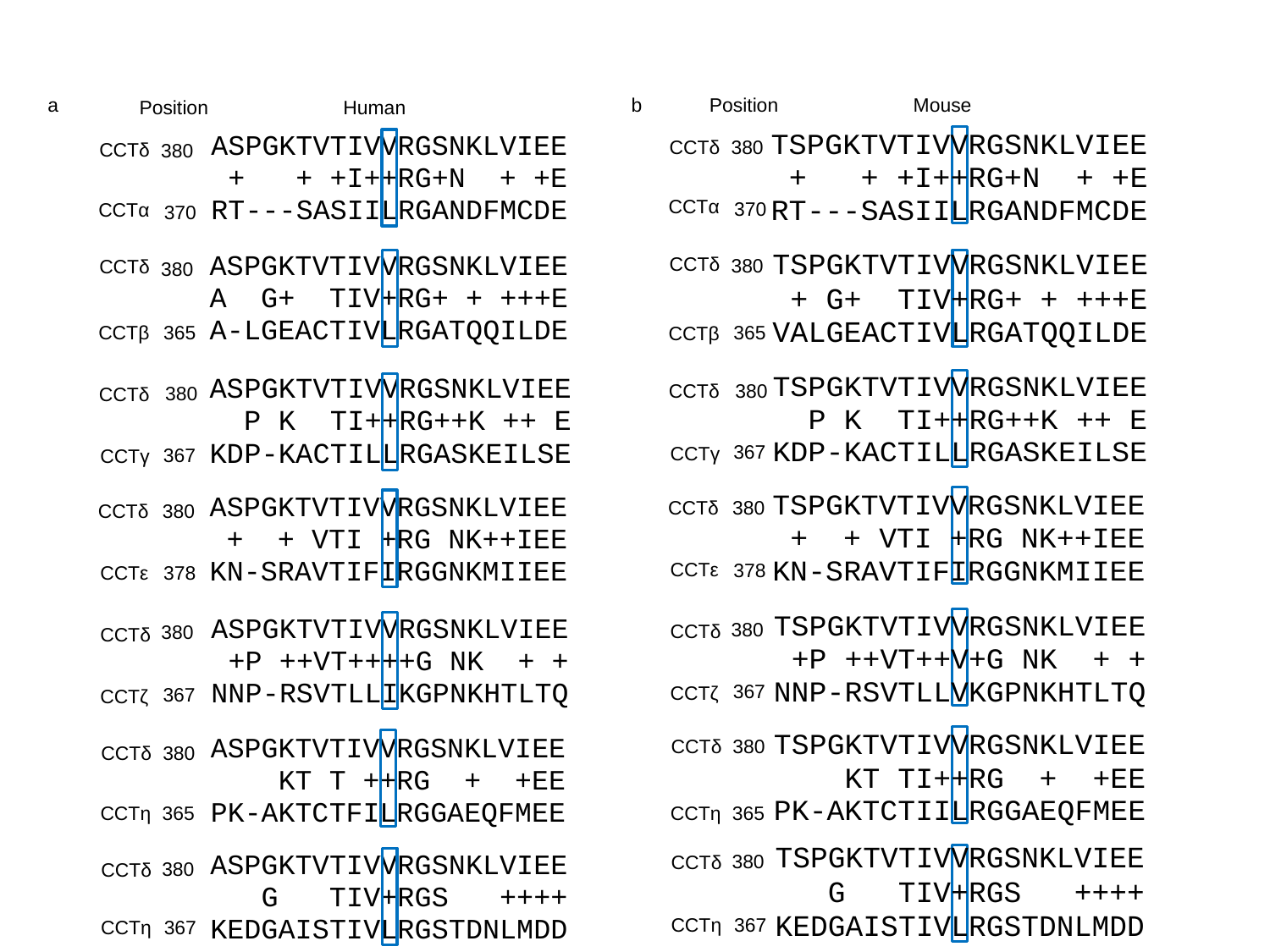

a
b
Position	 Mouse
CCTδ
380
CCTα
370
CCTδ
380
365
CCTβ
380
CCTδ
367
CCTγ
380
CCTδ
CCTε
378
380
CCTδ
367
CCTζ
380
CCTδ
CCTη
365
380
CCTδ
CCTη
367
Position	 Human
CCTδ
380
CCTα
370
CCTδ
380
CCTβ
365
380
CCTδ
367
CCTγ
380
CCTδ
CCTε
378
380
CCTδ
367
CCTζ
380
CCTδ
CCTη
365
380
CCTδ
CCTη
367
