# Supplementary figures and images for: Functional assessment of the V390F mutation in the CCTδ subunit of chaperonin containing tailless complex polypeptide 1
Source: Cell Stress Chaperones. 2021 Oct 15;26(6):955–64. doi: 10.1007/s12192-021-01237-x (PMC8578507; doi:10.1007/s12192-021-01237-x)

## Slide 1
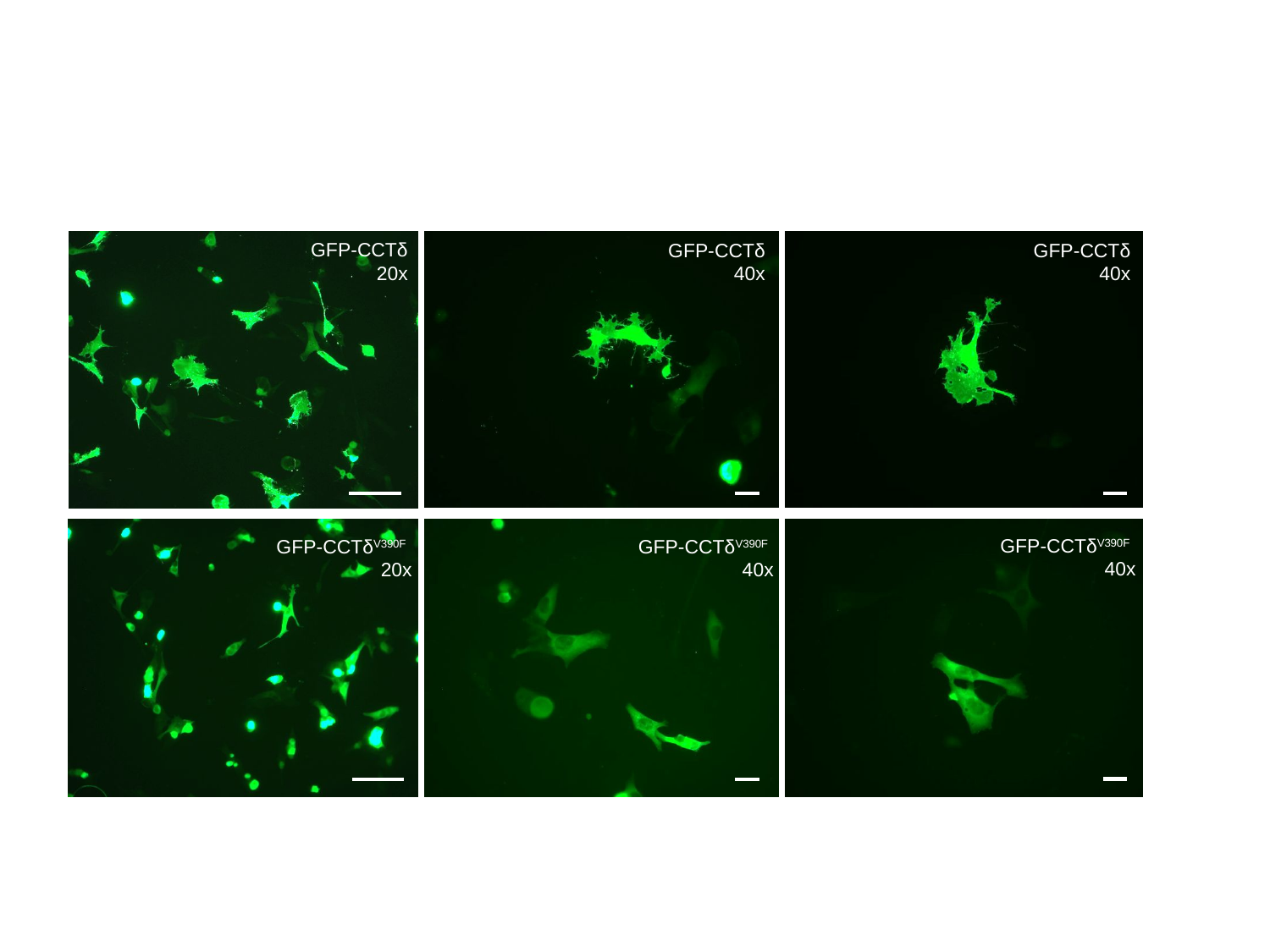

GFP-CCTδ
 20x
GFP-CCTδV390F
 20x
GFP-CCTδ
 40x
GFP-CCTδ
 40x
GFP-CCTδV390F
 40x
GFP-CCTδV390F
 40x

Supplement: Supplementary file 2 — Supplementary Fig. 2 Representative fields of view are shown of B16F1 cells transfected with either GFP-CCTδ or GFP-CCTδV390F. For both constructs one image taken at × 20 magnification (scale bar 100 μm) and two images taken at × 40 magnification (scale bar 10 μm) are shown. (PPTX 716 KB) [file 12192_2021_1237_MOESM2_ESM.pptx]
